# Association Between Sleep Disorders and Cognitive Impairment in Middle Age and Older Adult Hemodialysis Patients: A Cross-Sectional Study

**DOI:** 10.3389/fnagi.2021.757453

**Published:** 2021-12-08

**Authors:** Ru Tian, Yun Bai, Yidan Guo, Pengpeng Ye, Yang Luo

**Affiliations:** ^1^Division of Nephrology, Beijing Shijitan Hospital, Capital Medical University, Bejing, China; ^2^Department of Obstetrics and Gynecology, Beijing Jishuitan Hospital, Beijing, China; ^3^Division of Injury Prevention and Mental Health, National Center for Chronic and Non-communicable Disease Control and Prevention, Chinese Center for Disease Control and Prevention, Beijing, China

**Keywords:** sleep, sleep disorders, cognitive function, cognitive impairment, hemodialysis

## Abstract

**Objective:** The aims of the current study were to (1) explore the features of overall poor sleep and specific sleep disorders in Chinese middle age and older adult hemodialysis patients; (2) examine the association between sleep disorders and cognitive impairment (CI) in middle age and older patients undergoing hemodialysis in China.

**Methods:** Data of patients undergoing maintenance hemodialysis were collected from the prospective cohort study of CI in Chinese patients undergoing hemodialysis (Registered in Clinical Trials.gov, ID: NCT03251573). We included 613 patients (mean age = 63.7; *SD* = 7.8) in this study. We assessed sleep conditions using the Pittsburgh Sleep Quality Index (PSQI) questionnaire and cognitive function by the Chinese Beijing version of the Montreal Cognitive Assessment (MoCA-BJ) scale. Then the association between sleep disorders and CI was evaluated using multivariate logistic regression analysis.

**Results:** The prevalence of sleep disorders in this group of 613 hemodialysis patients was 77.0%. Patients with CI were more inclined to have sleep disorders in specific aspects of sleep latency, habitual sleep efficiency, sleep disturbances, and daytime dysfunction (*p* < 0.05). In multivariate logistic regression analyses, every 1-point increase in global PSQI score was associated with a 1.2-fold increased risk of CI (adjusted OR = 1.201; 95%CI = 1.123–1.284, *p* < 0.001). For each specific PSQI, every 1-point increase in sleep disturbances score was associated with a 2.6-fold increased risk of CI (adjusted OR = 2.624; 95%CI = 1.891–3.640, *p* < 0.001), and every 1-point increase in daytime dysfunction score was associated with a 3.7-fold increased risk of CI (adjusted OR = 3.709; 95%CI = 2.653–5.184, *p* < 0.001), whereas every 1-point increase in sleep duration score was associated with a decreased risk of CI (adjusted OR = 0.600; 95%CI = 0.434–0.830, *p* = 0.002).

**Conclusion:** Poor sleep quality especially sleep disturbances, daytime dysfunction, and long sleep duration are associated with CI in middle age and older adult hemodialysis patients. Thus, the early detection of sleep disorders may help identify patients with cognitive impairment among hemodialysis individuals.

**Clinical Trial Registration:** [Clinical Trials.gov], identifier [NCT03251573]

## Introduction

Cognitive impairment (CI) is common in individuals with chronic kidney disease (CKD), especially in hemodialysis patients with end-stage renal disease (ESRD) (Murray et al., 2006). Previous studies including our own indicated that the prevalence of CI was ranged from 70 to 80%, which was significantly higher than those individuals with normal kidney function (Kurella Tamura et al., 2010; O’Lone et al., 2016; van Zwieten et al., 2018; Luo et al., 2020). Besides, CI was proved to be associated with adverse outcomes by means of influencing the compliance of hemodialysis patients regarding their dialysis schedules and medication regimens (Drew et al., 2015; Angermann et al., 2018). Facing such a great challenge, it seems critically important to make further exploration about the detection and prevention of CI among hemodialysis patients.

Sleep quality, another common public health issue, has been proved to be related to poor cognitive function in the general population (Ratcliff and Van Dongen, 2009). Researchers demonstrated that disturbed sleep, including sleep fragmentation, abnormal sleep duration, and sleep disorders, were independent risk factors for CI among those populations (Waters and Bucks, 2011; Gadie et al., 2017). Notably, sleep disorders are also shown to be highly prevalent in hemodialysis and peritoneal dialysis patients, the prevalence of sleep disorder was approximately 49.0 to 60.5% among those individuals (Kutner et al., 2007; Elder et al., 2008; Davison et al., 2015), and this deterioration generally covered a wide range of aspects including sleep satisfaction, timing, efficiency, and duration, etc (Buysse, 2014). Acting as two major problems which may have a negative influence on the quality of life among dialysis patients, the relationship between sleep disorder and CI has caused attention in recent years, however, the results of the relationship were inconsistent (Rodriguez et al., 2013; Zhao et al., 2019). A recent study in peritoneal dialysis patients noted that possible narcolepsy was associated with general cognitive dysfunction (Zhao et al., 2019), and another study concluded that sleep-disordered breathing was associated with CI, in patients with advanced CKD (Kang et al., 2012). In contrast with those studies, Rodriguez et al. (2013) reported no association between sleep disturbances and cognitive function in hemodialysis patients. These different results may be related to the difference in accessing methods of sleep and cognitive function and different individuals in those studies. Further exploration about the relationship between them is in great need in hemodialysis patients.

To clarify this important issue, we conducted a cross-sectional analysis to assess the features of sleep status in a group of middle age and older hemodialysis patients using the Pittsburgh Sleep Quality Index (PSQI) questionnaire and assess cognitive function by the Chinese Beijing version of the Montreal Cognitive Assessment (MoCA-BJ) scale, then we evaluated the association between overall and specific sleep disorders with CI.

## Materials and Methods

### Study Design and Participants

This cross-sectional study used the data repository from the prospective cohort study of CI in Chinese patients undergoing hemodialysis (Registered in Clinical Trials.gov, ID: NCT03251573). Potential study participants were maintenance hemodialysis patients recruited from 11 hemodialysis centers in Beijing, who were screened for eligibility between April 2017 and June 2017. The eligibility criteria were as follows: (1) aged 50 to 80 years, (2) diagnosed with ESRD and treated with regular hemodialysis for a minimum of 3 months, (3) willing to provide written informed consent, (4) ability to complete a 15 min cognitive test and a 10 min test of sleep quality, and (5) the patient’s first language was Chinese. The exclusion criteria were: (1) unable to participate for reasons such as sensory (e.g., visual and hearing) or motor impairment, and (2) recently diagnosed with major psychiatric disorders (e.g., psychosis and depression). All participants gave their written informed consent before they were included in the study. This study was conducted under the ethical standards described by the Declaration of Helsinki and approved by the Institutional Ethical Review Board of Beijing Shijitan Hospital, Capital Medical University (approval no. SJT2016-18), this approved certificate in the principal investigator’s hospital was also authorized by other joining hospitals as a general ethical document.

### Assessment of Sleep Conditions

The sleep quality of each participant was self-assessed using the PSQI questionnaire, covering seven components of sleep quality (subjective sleep quality, sleep latency, sleep duration, habitual sleep efficiency, sleep disturbances, sleeping medication use, and daytime dysfunction) in the preceding month (Buysse et al., 1989). The PSQI questionnaire contained 19 self-rated questions and 5 questions rated by the bed partner or roommate (if one was available). Only self-rated questions were included in the scoring. The 19 self-rated items were combined to form seven “component” scores, each of which had a score ranged from 0 to 3, and a score of 0 indicated no difficulty, while a score of 3 indicated severe difficulty. The seven component scores were then added to yield one “global” score, ranged from 0 to 21. Higher scores indicated poorer overall sleep quality. A global score of <5, 5–10, 11–15, and >15 indicated good, fairly good, fairly bad, and bad. In general, a global PSQI score of >5 indicated poor sleep quality (Buysse et al., 1989). We gave special training to the relatives or caregivers for some special patients who were not able to make self-reports about the sleep quality. The assessment was conducted on the day after a dialysis session and required approximately 5–10 min.

### Neuropsychological Assessment and Cognitive Impairment Diagnosis

Cognitive function was assessed by the MoCA-BJ scale (Lu et al., 2011; Wang et al., 2014). The scale consisted of seven components: visuospatial and executive function, naming, memory, attention, language, abstraction, and orientation. The scores ranged from 0 to 30. A score of <26 suggested CI and those who were educated less than 12 years were added 1 point to correct for educational deviation. All the tests were conducted under the direction of the staff who were trained by the neuropsychologists before the study. The assessment was conducted on the day after a dialysis session and required on average approximately 15 min. Depression was assessed using the Hamilton Depression Scale, with scores ranging from 0 to 63, and a score of ≥7 suggested suspected depression (Eggers et al., 2003).

### Statistical Analysis

Data were presented as the mean ± standard deviation (SD) for continuous variables with normal distribution, medians and interquartile ranges for those with non-normal distribution, and proportions for categorical variables. Student’s t-tests, Mann-Whitney tests, and Chi-square tests were used to compare the demographics, clinical characteristics, the global and seven component sleep scores stratified by the level of cognitive function.

We used multivariate logistic regression analysis to evaluate sleep conditions (global score and seven component scores, respectively) associated with CI or not. Variables associated with CI on unadjusted analyses with *p* values ≤ 0.10 and potential clinical risk factors for CI were entered into the logistic regression model as covariates, with CI as the dependent variables, adjusted for age, sex, education level, smoking history, alcohol intake, comorbidities including the medical history of diabetes, hypertension, stroke, coronary heart disease, stroke, and depression status, hemodialysis vintage, single-pool Kt/V, and the serum levels of hemoglobin (Hb), albumin (Alb), and intact parathyroid hormone (iPTH). All analyses were conducted with SPSS v.21 (IBM Corp., Chicago, IL, United States), using two-tailed 95% confidence intervals (CI), *p* values < 0.05 were considered statistically significant.

## Results

### Demographics and Clinical Characteristics of the Patients

We enrolled 613 patients in this study, the mean age was 63.7 ± 7.8 years, 42.1% were women, and only 6.2% had less than 6 years of education. Hypertension (88.9%), diabetes (37.7%), and coronary heart disease (31.5%) were the most common diseases in medical history. Each average dialysis treatment session was 3.8 ± 0.3 h. The more detailed demographics and clinical characteristics of the patients were shown in Table 1 according to the CI group or normal cognitive function (NCF) group. The prevalence of CI in this study was 80.9%. Patients with CI were more likely to have a lower education level, comorbidities of diabetes, hypertension, and stroke, a longer hemodialysis vintage, a lower level of single-pool Kt/V, and lower MoCA-BJ scores.

**TABLE 1:** Demographics and clinical characteristics of the study population.

Variables	Total (*n* = 613)	NCF (*n* = 117)	CI (*n* = 496)	*p* value
Age (years)	63.7 ± 7.8	59.3 ± 7.7	64.7 ± 7.4	<0.001
Gender, female	258 (42.1%)	47 (40.2%)	211 (42.5%)	0.641
**Education level**				0.001
<6 years	38 (6.2%)	6 (5.1%)	32 (6.5%)	
6–12 years	406 (66.2%)	63 (53.8%)	343 (69.2%)	
>12 years	169 (27.6%)	48 (41.0%)	121 (24.4%)	
**Smoking history**				0.925
Never	343 (56.0%)	66 (56.4%)	277 (55.8%)	
Former	186 (30.3%)	34 (29.1%)	152 (30.6%)	
Current	84 (13.7%)	17 (14.5%)	67 (13.5%)	
**Alcohol intake**				0.936
Never	352 (57.4%)	66 (56.4%)	286 (57.7%)	
Former	233 (38.0%)	45 (38.5%)	188 (37.9%)	
Current	28 (4.6%)	6 (5.1%)	22 (4.4%)	
Diabetes	231 (37.7%)	33 (28.2%)	198 (39.9%)	0.019
Hypertension	545 (88.9%)	95 (81.2%)	450 (90.7%)	0.003
Stroke	100 (16.3%)	6 (5.1%)	94 (19.0%)	<0.001
CHD	193 (31.5%)	36 (30.8%)	157 (31.7%)	0.853
BMI (kg/m^2^)	23.6 ± 4.1	24.2 ± 5.8	23.5 ± 3.6	0.202
Dialysis vintage (mo.)	57.0 (24.0, 102.0)	42.0 (12.0, 78.0)	60.0 (29.0, 106.0)	<0.001
Single-pool Kt/V	1.3 ± 0.2	1.3 ± 0.2	1.2 ± 0.2	0.002
Hb (g/L)	111.4 ± 14.7	111.3 ± 15.3	111.5 ± 14.5	0.914
Alb (g/L)	40.3 ± 3.0	40.6 ± 2.3	40.2 ± 3.1	0.088
Calcium (mmol/L)	2.2 ± 0.2	2.2 ± 0.2	2.2 ± 0.3	0.127
Phosphate (mmol/L)	1.7 ± 0.7	1.8 ± 0.7	1.7 ± 0.6	0.342
iPTH (pg/mL)	189.0 (103.0, 357.5)	162.8 (101.5, 295.8)	204.3 (103.0, 368.7)	0.058
MoCA-BJ score	23.9 ± 3.5	27.1 ± 1.3	21.9 ± 2.8	<0.001
Depression score	4.8 ± 5.1	5.2 ± 5.5	4.77 ± 5.0	0.287

*NCF, normal cognitive function; CI, cognitive impairment; CHD, coronary heart disease; BMI, body mass index; Kt/V, an indicator for evaluating dialysis adequacy; Hb, hemoglobin; Alb, albumin; iPTH, intact parathyroid hormone; MoCA-BJ, the Chinese Beijing version of the Montreal Cognitive Assessment.*

### Comparison of Sleep Quality in Patients With Different Cognitive Function Levels

The prevalence of sleep disorders in this study was 77.0%. The global and seven component scores of PSQI in patients were shown in Table 2. Patients with CI were more inclined to have sleep disorders in global PSQI and specific components including sleep latency, habitual sleep efficiency, sleep disturbances, and daytime dysfunction.

**TABLE 2:** Global and seven component scores of PSQI, in HD patients with NCF and CI.

Component (scores)	Total (*n* = 613)	NCF (*n* = 117)	CI (*n* = 496)	*p* value
Subjective sleep quality	0.0 (0.0, 1.5)	0.0 (0.0, 1.5)	1.0 (0.0, 1.8)	0.659
Sleep latency	1.0 (0.0, 2.0)	1.0 (0.0, 2.0)	1.0 (1.0, 2.0)	<0.001
Sleep duration	1.0 (0.0, 3.0)	1.0 (0.0, 2.0)	1.0 (0.0, 3.0)	0.220
Habitual sleep efficiency	2.0 (1.0, 2.0)	1.0 (0.0, 2.0)	2.0 (1.0, 2.0)	0.016
Sleep disturbances	1.0 (0.0, 2.0)	0.0 (0.0, 1.0)	1.0 (0.0, 3.0)	<0.001
Use of sleeping medication	1.0 (1.0, 1.0)	1.0 (1.0, 1.0)	1.0 (1.0, 1.0)	0.175
Daytime dysfunction	3.0 (1.0, 3.0)	1.0 (1.0, 1.0)	3.0 (2.0, 3.0)	<0.001
Global PSQI	9.0 (6.0, 13.0)	6.0 (4.0, 10.0)	10.0 (6.0, 14.0)	<0.001

*PSQI, the Pittsburgh Sleep Quality Index; HD, hemodialysis; NCF, normal cognitive function; CI, cognitive impairment.*

**FIGURE 1:**
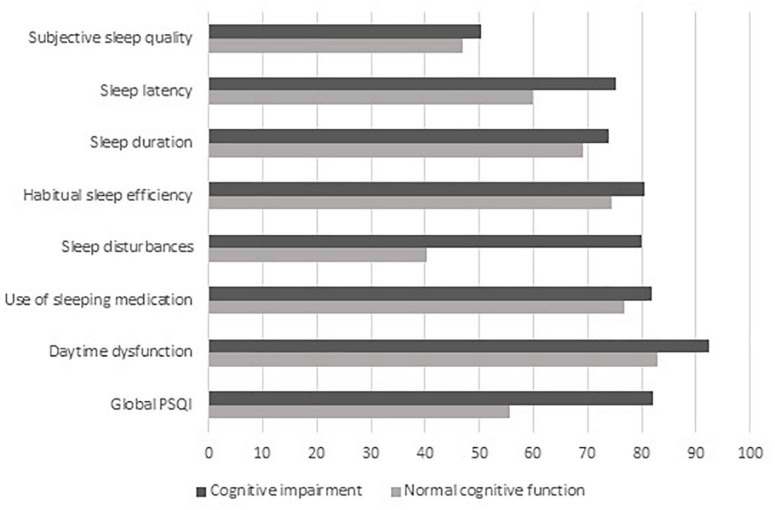
Proportion of a score of >5 on global and a score of 3 on each specific PSQI in HD patients with or without CI. PSQI, the Pittsburgh Sleep Quality Index; HD, hemodialysis; CI, cognitive impairment.

The proportion of a score of >5 on global and a score of 3 on each specific PSQI in patients was illustrated in Figure 1, stratified according to the cognitive function. Compared to patients with NCF, the proportion on global and each specific PSQI in patients with CI was seemed to be higher (82.1% of CI, and 55.6% of NCF on global PSQI, 50.4% of CI, and 47.0% of NCF on subjective sleep quality, 75.2% of CI, and 59.8% of NCF on sleep latency, 74.0% of CI, and 69.2% of NCF on sleep duration, 80.4% of CI, and 74.4% of NCF on habitual sleep efficiency, 80.0% of CI, and 40.2% of NCF on sleep disturbances, 81.9% of CI, and 76.9% of NCF on use of sleeping medication, and 92.3% of CI, and 82.9% of NCF on daytime dysfunction, respectively).

### Association Between Sleep Disorders and Cognitive Impairment

The association between overall poor sleep (global PSQI score) with CI was shown in Table 3. In the fully adjusted logistic regression model, every 1-point increase in global PSQI score was associated with a 1.2-fold increased risk of CI (adjusted OR = 1.201; 95%CI = 1.123–1.284). We also investigated whether each specific sleep disorder (specific PSQI score) was associated with CI, for these seven components, only sleep disturbances, daytime dysfunction, and sleep duration were associated with CI. Every 1-point increase in sleep disturbances score was associated with a 2.6-fold increased risk of CI (adjusted OR = 2.624; 95%CI = 1.891–3.640), and every 1-point increase in daytime dysfunction score was associated with a 3.7-fold increased risk of CI (adjusted OR = 3.709; 95%CI = 2.653–5.184), whereas every 1-point increasing in sleep duration score was associated with a decreased risk of CI (adjusted OR = 0.600; 95%CI = 0.434–0.830).

**TABLE 3:** Logistic regression for the association between overall and specific sleep disorders (global and specific PSQI scores) with cognitive impairment.

Variables	Crude		Adjusted	
	OR (95%CI)	*p* value	OR (95%CI)	*p* value
Global PSQI*	1.188 (1.127–1.253)	<0.001	1.201 (1.123–1.284)	<0.001
Specific PSQI*, subjective sleep quality	1.025 (0.848–1.238)	0.801	0.925 (0.675–1.268)	0.626
Sleep latency	1.426 (1.169–1.740)	<0.001	0.857 (0.549–1.337)	0.496
Sleep duration	1.118 (0.934–1.337)	0.224	0.600 (0.434–0.830)	0.002
Habitual sleep efficiency	1.267 (1.042–1.539)	0.018	0.827 (0.561–1.218)	0.336
Sleep disturbances	2.022 (1.634–2.502)	<0.001	2.624 (1.891–3.640)	<0.001
Use of sleeping medication	1.307 (0.886–1.930)	0.177	1.146 (0.565–2.325)	0.705
Daytime dysfunction	3.109 (2.475–3.905)	<0.001	3.709 (2.653–5.184)	<0.001

*Adjusted OR calculated with logistic regression, with adjustment for age, sex, education level, smoking history, alcohol intake, comorbidities, hemodialysis vintage, Kt/V, the serum level of Hb, ALB, and iPTH and depression score in each model, respectively. PSQI, the Pittsburgh Sleep Quality Index; OR, odds ratio; CI, confidence interval; Kt/V, an indicator for evaluating dialysis adequacy; Hb, hemoglobin; ALB, albumin; iPTH, intact parathyroid hormone.

## Discussion

Our study showed that the prevalence of sleep disorders in this group of Chinese middle age and older adult hemodialysis patients was 77.0%, the prevalence of CI in those patients was 80.9%. Patients with CI were more inclined to have sleep disorders in specific aspects of sleep latency, habitual sleep efficiency, sleep disturbances, and daytime dysfunction. These results remained almost consistent in following multivariate adjustment, suggesting that poor sleep quality especially sleep disturbances, daytime dysfunction, and long sleep duration were associated with CI in middle age and older adult hemodialysis patients.

Currently, both sleep disorders and CI have become serious public health issues in aging and increase with advancing age (Gadie et al., 2017). Our study found the prevalence of sleep disorders was 77.0%, which was almost in agreement with previous data (45.6–88.5%) in hemodialysis patients (Iliescu et al., 2003; Bastos et al., 2007; Pai et al., 2007; Elder et al., 2008; Eryavuz et al., 2008; Cengic et al., 2012; Harris et al., 2012). The relatively wide range of reported prevalence may be related to different individuals and the difference in accessing methods of sleep quality in the studies. A study including 128 American hemodialysis patients reported the prevalence of sleep disorders was 45.6%, which was lower than that in our study, this might be related to the different regional characteristics and ages of the participants (the mean age was 57.3 ± 13.8 years in Harris’s study and 63.7 ± 7.8 years in our study) (Harris et al., 2012). Another study including 11,351 hemodialysis patients from 7 countries found nearly half (49%) of patients experienced sleep disorders, in which they accessed sleep quality through a patient self-reported scale while not the PSQI questionnaire (Elder et al., 2008). In recent years, the PSQI questionnaire has been widely used and recognized as a well-established instrument of self-reported sleep quality among older adults in different countries (Beaudreau et al., 2012; Hinz et al., 2017; Salahuddin et al., 2017). Therefore, it is vital to establish consistent methods for assessing sleep quality and we recommend using the PSQI questionnaire to improve the accuracy and reliability of the diagnosis of sleep disorders in hemodialysis patients.

In addition, our analysis and previous studies also indicated that CI was very common and related to poor outcomes in hemodialysis patients, the increased age, lower education level, cardiovascular and cerebrovascular diseases, and dialysis-related factors including hemodialysis vintage and single-pool Kt/V were shown to be the independent risk factors of CI (Kurella Tamura et al., 2010; Drew et al., 2015; O’Lone et al., 2016; Luo et al., 2020). As a major public health issue, sleep disorders are shown to be associated with CI in community individuals (Gadie et al., 2017). One of the largest studies including 3,151 Japanese community-dwelling older individuals revealed that both long sleep duration and excessive daytime sleepiness were independent risk factors for cognitive decline after 4 years of follow-up (Nakakubo et al., 2019). However, as we mentioned above, the association between sleep disorders and CI is still in controversy concerning hemodialysis patients. Kutner et al. (2007) investigated the relationship of sleep difficulty and cognitive function scores in a national cohort of 2,286 dialysis patients, the authors noted that patients with lower cognitive scores were more likely to report sleep difficulty, and the association also was significant in a subgroup analysis that was restricted to hemodialysis patients. While Rodriguez et al. (2013) reported no association between sleep disturbances and cognitive function in 168 hemodialysis patients, they applied the sleep subscale battery of the Choices for Healthy Outcomes in Caring for ESRD to assess sleep disturbances and a detailed battery of neurocognitive tests to assess cognitive function. The differences of the above mentioned results may be related to the possibility that both the sleep questionnaire and the cognitive function battery are not consistent and sensitive enough to appreciate this association accurately (Kurella et al., 2004; Rodriguez et al., 2013). In our study, sleep disorders were assessed through the PSQI questionnaire and cognitive function by the MoCA-BJ scale. Currently, the PSQI questionnaire has been recognized as an internally consistent and valid measure of self-reported sleep quality among older adults in a variety of languages and clinical circumstances (Beaudreau et al., 2012; Hinz et al., 2017; Salahuddin et al., 2017). The MoCA-BJ scale is a convenient assessing tool for cognitive function and we have validated its sensitivity and specificity with the fifth version of the Diagnostic and Statistical Manual of Mental Disorders (DSM-V) recommended neuropsychological battery (Tian et al., 2020). Through these two widely accepted evaluation tools, we found that sleep disorders especially sleep disturbances, daytime dysfunction, and long sleep duration were significantly associated with CI in middle age and older adult hemodialysis patients. The association between them provided a useful hint that we should pay more attention to the evaluation of both sleep and cognitive function among hemodialysis patients in the clinical practice, and it seems that sleep function improvement might be benefitial in retarding the development of CI among those individuals.

Our study has several limitations. First, the study was a cross-sectional analysis, we could not determine the causal relationship between sleep disorders and CI. Second, the sleep scale in our study was self-reported and this might lead to a reporting bias. Even with the above-mentioned limitations, we still have several strengths concerning our study. First, we included a large cohort of 613 patients selected from 11 hemodialysis centers in Beijing which might have a good representative of hemodialysis patients in China. Second, sleep quality was assessed through the PSQI questionnaire and cognitive function by the MoCA-BJ scale, which might improve the sensitivity and accuracy of the diagnosis of sleep disorders and CI in hemodialysis patients.

## Conclusion

In summary, our results indicate that sleep disorders are associated with cognitive impairment in Chinese middle age and older adult hemodialysis patients. Identifying one of these issues might be a hint for the existence of another issue in hemodialysis patients, the data may be helpful for the clinician in the detection and prevention of these two important diseases in those individuals. Future studies should evaluate this relationship in prospective cohort studies with more detailed and objective measurements of sleep quality.

## Data Availability Statement

The raw data supporting the conclusions of this article will be made available by the authors, without undue reservation.

## Ethics Statement

The studies involving human participants were reviewed and approved by the Institutional Ethical Review Board of Beijing Shijitan Hospital, Capital Medical University (approval no. SJT2016-18). The patients/participants provided their written informed consent to participate in this study.

## Author Contributions

RT, YB, and YG collected data and contributed to the critical revision of the manuscript. RT, YG, and YL contributed to the hypothesis and study design and interpreted the result. RT and PY analyzed the data. RT wrote the manuscript. All authors contributed to the article and approved the submitted version.

## Conflict of Interest

The authors declare that the research was conducted in the absence of any commercial or financial relationships that could be construed as a potential conflict of interest. The reviewer XL has declared a shared parent affiliation with the authors RT, YG, and YL at the time of the review.

## Publisher’s Note

All claims expressed in this article are solely those of the authors and do not necessarily represent those of their affiliated organizations, or those of the publisher, the editors and the reviewers. Any product that may be evaluated in this article, or claim that may be made by its manufacturer, is not guaranteed or endorsed by the publisher.
