# Corrigendum: Association Between Sleep Disorders and Cognitive Impairment in Middle Age and Older Adult Hemodialysis Patients: A Cross-Sectional Study

**DOI:** 10.3389/fnagi.2021.831897

**Published:** 2022-01-05

**Authors:** Ru Tian, Yun Bai, Yidan Guo, Pengpeng Ye, Yang Luo

**Affiliations:** ^1^Division of Nephrology, Beijing Shijitan Hospital, Capital Medical University, Bejing, China; ^2^Department of Obstetrics and Gynecology, Beijing Jishuitan Hospital, Beijing, China; ^3^Division of Injury Prevention and Mental Health, National Center for Chronic and Non-communicable Disease Control and Prevention, Chinese Center for Disease Control and Prevention, Beijing, China

**Keywords:** sleep, sleep disorders, cognitive function, cognitive impairment, hemodialysis

In the published article, there was an error regarding the co-author affiliation. Instead of “Ru Tian^1^, Yun Bai^2^”, it should be “Ru Tian^1+^, Yun Bai^2+^” with the note “^+^These authors have contributed equally to this work and share the first authorship”.

The authors apologize for this error and state that this does not change the scientific conclusions of the article in any way. The original article has been updated.

## Publisher's Note

All claims expressed in this article are solely those of the authors and do not necessarily represent those of their affiliated organizations, or those of the publisher, the editors and the reviewers. Any product that may be evaluated in this article, or claim that may be made by its manufacturer, is not guaranteed or endorsed by the publisher.

